# Pharmacological interventions for obesity in patients with inflammatory bowel disease: A systematic review of GLP-1 receptor agonist efficacy and safety

**DOI:** 10.1016/j.obpill.2026.100272

**Published:** 2026-04-28

**Authors:** Asif Jan, Daniel Dubwa

**Affiliations:** a District Headquarter Hospital, Charsadda, PO.Box 24430, KP, Pakistan; b Saidu Group of Teaching Hospital, Saidu Sharif, Swat, PO.Box 19200, KP, Pakistan; c Department of Pharmacy, Makerere University, PO.Box 10008, Kampala, Uganda

**Keywords:** Inflammatory Bowel Disease (IBD), Obesity, Glucagon-like peptide-1 receptor agonists, Weight loss

## Abstract

**Background:**

Obesity is an escalating global health challenge, particularly among patients with inflammatory bowel disease (IBD), where the coexistence of metabolic dysfunction and chronic intestinal inflammation complicates clinical management. Glucagon-like peptide-1 receptor agonists (GLP-1 RAs) are potent weight-management agents; however, their efficacy and safety profiles in patients with obesity and IBD specifically regarding gastrointestinal tolerability remain poorly characterized.

**Methods:**

We conducted a systematic review to evaluate the impact of GLP-1 RAs on anthropometric outcomes in patients with obesity and IBD. A comprehensive search of PubMed, Embase, and Web of Science was performed through November 10, 2024. Observational studies reporting weight-related metrics were synthesized. To ensure scientific rigor, all imperial units were converted to metric (kg), and a narrative synthesis was employed to address heterogeneity in study designs.

**Results:**

A total of n = 7 studies (sample sizes from 16 to 47,424 participants) were analyzed. GLP-1 RAs consistently demonstrated significant weight reduction, with reported losses ranging from a median of −6.2 kg (range: −3.4 to −8.5 kg) to a mean of approximately −9.5 kg (SD = 8.5) over follow-up periods of 3–18 months. Greater weight loss was observed in patients with higher baseline body mass index and longer treatment duration. Overall, reductions ranged from approximately −16 lbs (SD = 13) to −26 lbs (SD = 20), with longer follow-up studies (up to 450 days) showing median reductions of −8.15 kg (range: −15.9 to −2.2 kg).

**Conclusions:**

GLP-1 RAs are effective pharmacological adjuncts for weight loss in patients with obesity and IBD, with outcomes modulated by treatment duration and agent selection. These findings support the clinical utility of incretin-based therapies in this complex patient population. However, large-scale randomized controlled trials are essential to establish long-term safety and determine the impact of these agents on IBD disease activity.

## Highlights

• GLP-1 receptor agonists (GLP-1 RAs) demonstrate consistent and clinically significant weight loss in patients with obesity and inflammatory bowel disease (IBD), with reported reductions ranging from approximately −6.2 kg to −9.5 kg, and even greater reductions observed in studies with extended follow-up durations.
• The magnitude of weight loss appears to be influenced by treatment duration and baseline patient characteristics, particularly body mass index (BMI), with patients having higher baseline BMI and longer exposure to therapy experiencing more pronounced and sustained weight reduction.
• Among the different GLP-1 RAs evaluated, semaglutide and tirzepatide show comparatively greater efficacy in promoting weight loss, suggesting potential differences in pharmacological potency and mechanisms of action across agents.
• Adverse events, including nausea, diarrhea, vomiting, and constipation, are the most frequently reported side effects; however, these are generally mild to moderate in severity and manageable with appropriate clinical strategies such as dose titration.
• Importantly, current evidence does not indicate a significant increase in IBD exacerbations or disease worsening, supporting the relative safety of GLP-1 RAs in this population, although further large-scale, long-term randomized studies are needed to confirm these findings and guide clinical practice.

## Graphical abstract

**Figure:**
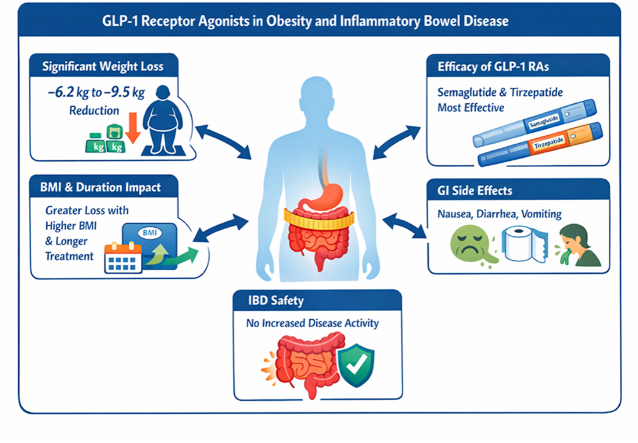

## 1 Introduction

Obesity represents an escalating global health priority, with its prevalence increasing markedly on a worldwide scale [1]. According to the World Health Organization (WHO), approximately 40% of the adult population was classified as overweight and 13% as having obesity as of 2016 [1,2]. The condition is linked to a range of comorbidities, including type 2 diabetes, cardiovascular diseases, and metabolic disorders, contributing to millions of deaths annually [3,4]. Inflammatory Bowel Disease (IBD) is a chronic condition characterized by inflammation of the gastrointestinal tract, leading to symptoms such as abdominal pain, diarrhea, and weight loss [5]. IBD affects approximately 7 million people globally [6]. The two main types of IBD are Crohn's disease and ulcerative colitis, each with distinct patterns of inflammation and varying clinical presentations [6]. Interestingly, patients with IBD are at risk for both malnutrition and obesity. Approximately 15–40% of individuals with IBD are overweight [7], complicating disease management and exacerbating overall health outcomes.

Recently, the clinical management of obesity in patients with IBD has become a focal point of research, driven by the unique challenges of metabolic control within an inflammatory context. Beyond traditional nutritional interventions and bariatric surgery, novel pharmacological strategies have gained prominence, specifically glucagon-like peptide-1 receptor agonists (GLP-1 RAs) [8]. Although originally engineered for glycemic control in type 2 diabetes, GLP-1 RAs have evolved into a transformative therapeutic class for weight reduction across diverse clinical cohorts [8]. These agents mimic the natural hormone GLP-1, which regulates glucose metabolism and appetite [9]. GLP-1 RAs promote weight loss by reducing appetite, increasing satiety, and slowing gastric emptying, which collectively lead to reduced caloric intake [10]. In clinical trials, GLP-1 RAs have been shown to reduce body weight by an average of 17% in patients with obesity [11]. Additionally, these drugs improve glucose control by enhancing insulin secretion in a glucose-dependent manner [12], further supporting weight management.

The potential of GLP-1 RAs to promote weight loss in IBD patients is of particular interest, as excessive weight can exacerbate comorbidities associated with both obesity and IBD. However, concerns have been raised about the safety of these drugs in this patient group, as IBD is characterized by chronic inflammation of the gastrointestinal tract [13]. The use of GLP-1 RAs, which can cause gastrointestinal side effects such as nausea, vomiting, and abdominal discomfort, may pose risks in patients with IBD, potentially leading to exacerbation of disease symptoms or new complications. A study on GLP-1 RA use in general populations found that 15–20% of patients experienced mild to moderate gastrointestinal symptoms [14], raising further concerns about the safety profile in IBD patients. Moreover, the complex interplay between obesity, IBD, and other comorbidities means that the efficacy and safety of GLP-1 RAs in this patient group need thorough investigation.

Although numerous studies have explored the effects of GLP-1 RAs on weight loss in patients with metabolic disorders, there is a limited body of research specifically examining their impact in the patients with obesity and IBD. Findings from the available studies have been inconsistent. Some studies report substantial weight loss and favourable improvements in metabolic parameters, indicating that GLP-1 RAs could be a promising approach for obesity management in IBD patients [15]. However, other research raises concerns about the gastrointestinal side effects associated with GLP-1 RAs, including nausea, vomiting, and abdominal discomfort [16,17], which could potentially worsen IBD symptoms. These contradictory findings emphasize the necessity for more comprehensive research to better define the safety and effectiveness of GLP-1 RAs in IBD patients. As interest in using these agents for weight management in IBD grows, a clearer understanding of their benefits and risks is crucial for guiding clinical decision-making.

This systematic review sought to evaluate the clinical evidence regarding the efficacy and safety of GLP-1 RAs for weight reduction in patients with obesity and IBD. The primary objective is to clarify the therapeutic role of these agents within the IBD management paradigm and to provide evidence-based insights that inform clinical practice guidelines for patients presenting with this dual diagnosis.

## 2 Methods

### 2.1 Study design

This systematic review was conducted to synthesize existing evidence on the efficacy and safety of GLP-1 RAs for weight management in patients with obesity and IBD. The study protocol adhered to the Preferred Reporting Items for Systematic Reviews and Meta-Analyses (PRISMA) statement [18] (Table S1).

### 2.2 Data sources

A systematic search was executed across three electronic databases PubMed, Embase, and Web of Science to identify relevant literature published through Nov 20, 2024. These databases were selected for their comprehensive indexing of biomedical research related to gastroenterology and pharmacological interventions.

### 2.3 Search strategy

The search strategy employed a combination of Boolean operators and keywords including (“GLP-1 receptor agonists” OR “GLP-1 RAs” OR “liraglutide” OR “semaglutide” OR “exenatide” OR “dulaglutide”) AND (“inflammatory bowel disease” OR “IBD” OR “Crohn's disease” OR “ulcerative colitis”). No language or publication type restrictions were applied to ensure a comprehensive retrieval. The detailed search string is provided in Table S2.

### 2.4 Eligibility criteria

Studies were selected based on pre-defined inclusion and exclusion criteria. Inclusion criteria comprised [1]: observational studies (cohort, case-control, or cross-sectional) evaluating GLP-1 RA therapy in patients with obesity and IBD [2]; studies reporting quantitative weight-related outcomes, such as changes in Body Mass Index (BMI) or total body weight; and [3] research published in peer-reviewed journals. All GLP-1 RA classes and administration routes (injectable and oral) were considered eligible. Exclusion criteria included [1]: pediatric populations or patients with non-IBD gastrointestinal conditions [2]; studies lacking objective weight-loss data; and [3] case reports, conference abstracts, editorials, and review articles.

### 2.5 Study selection

Initial screening of titles and abstracts was performed independently by two reviewers against the eligibility criteria. Full-text articles were subsequently retrieved and evaluated for final inclusion. Discrepancies were resolved via internal consensus or consultation with a third reviewer. Semi-automated software (Nested-Knowledge, MN, USA) was utilized to identify and remove duplicate records and facilitate the screening process.

### 2.6 Data extraction and quality assessment

Data extraction was performed using a standardized extraction form that systematically captured key study characteristics, including the year of publication, author details, study location, sample size, research design, and participant demographics (e.g., age and sex). In addition, outcome data pertinent to the study's objectives were extracted, such as weight loss measures (e.g., changes in body mass index (BMI), percentage weight loss) and adverse events. Two reviewers independently conducted the extraction process to ensure accuracy and consistency. Any discrepancies between reviewers were resolved through discussion, and, if necessary, a third reviewer was consulted for adjudication. To optimize the efficiency and precision of the data extraction process, the “tagging” feature of the Nested-Knowledge platform was utilized to facilitate the organization, categorization, and streamlining of the data collection workflow.

Utilizing the Newcastle-Ottawa Scale (NOS), two assessors independently evaluated the quality of the included studies. The studies were assessed based on three key domains: participant selection, comparability of study groups, and the method of assessing the relationship between GLP-1 RAs treatment and weight loss or metabolic outcomes in patients with obesity and IBD (Table S3).

### 2.7 Data synthesis

A narrative synthesis approach was utilized to summarize the findings. A quantitative meta-analysis was not performed due to significant heterogeneity in study designs, diverse outcome measures, and varying follow-up durations across the included literature.

## 3 Results

### 3.1 Literature search

**Fig. 1:**
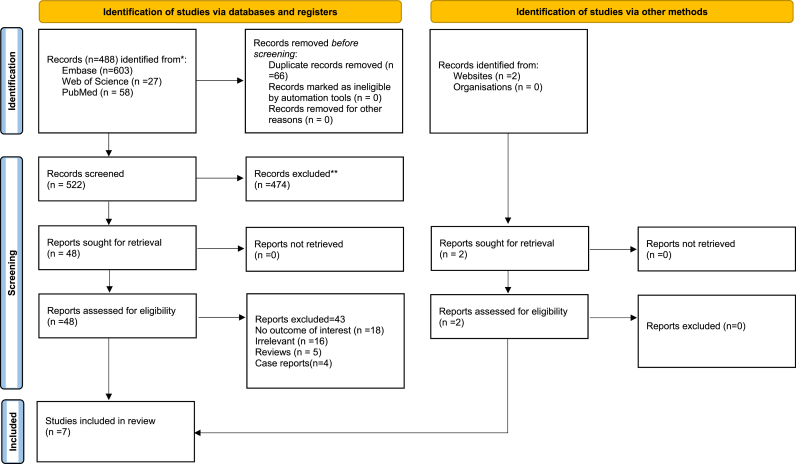
PRISMA flowchart depicting article selection and screening process.

A total of 588 entries were found through the database search, including 503 from Embase, 58 from PubMed, and 27 from Web of Science. Only 522 distinct records were left for screening after 66 duplicates were eliminated. This method left 48 full-text publications for in-depth evaluation after 474 records were eliminated because, according to their titles and abstracts, they did not match the inclusion requirements. After thorough analysis, 43 publications were disqualified for a variety of reasons, including case reports [4], review papers [5], irrelevant [18], and failure to address the outcomes of interest [16]. Finally,7 [[19], [20], [21], [22], [23], [24], [25]] studies were chosen to be part of the systematic review (Fig. 1).

### 3.2 Characteristics of included studies

The included studies, primarily conducted in the USA and Spain, comprised six retrospective cohorts and one case-control study (Table 1). Study populations were diverse, spanning various clinical presentations of IBD and obesity, with sample sizes ranging from 16 to 47,424 study participants. Participant ages ranged from 16 to over 60 years, with a predominant female representation of females. Follow-up durations varied from varied from 3 to 18 months.

**Table 1:** Systematic summary of studies included and clinical outcomes.

Study (Year)	Country/Design	Population (N)	Age/% Female	Baseline BMI/Weight	Weight/BMI Change	Adverse Events (n)
Belinchon 2024 [19]	Spain/RC	Obese IBD (16)	50y/81%	35 kg/m^2^/90.2 kg	ΔTBW: −6.2 kg	Nausea (2), Diarrhea (2)
Clarke 2024 [20]	USA/RC	IBD (169)	NA/60%	35.1 kg/m^2^/NA	ΔBMI: 3.45	Nausea (35), Diarrhea (10), Constipation (15)
					ΔTBW: −9.27 kg	
Desai 2024 [21]	USA/RC	IBD & Obesity (47,424)	47.4y/76%	36.9 kg/m^2^/237 lbs	ΔTBW: 16 to −26 kg	NA
Levine 2024 [22]	USA/RC	IBD (224)	54y/63%	−2.4 kg/m^2^ NA	End BMI: 31.6 kg	NA
Pham 2023 [23]	USA/CC	IBD & Obesity (72)	51.5y/NA	35.2 kg/m^2^/NA	ΔTBW: 1.8% to −9.8%kg	GI symptoms (6)
Sehgal 2024 [24]	USA/RC	Adult IBD (230)	58y/59%	NA/223 lbs	End TBW: 207 kg	Nausea (12), GI (6), Other (4)
St-Pierre 2024 [25]	USA/RC	Obese IBD (36)	45.5y/64%	34.0 kg/m^2^/92.5 kg	ΔTBW: −8.15 kg	Nausea (11), Constipation (9), Vomit (3)

**Footnotes**: Data represent a synthesis of retrospective and case-control studies across diverse geographical regions, highlighting variations in Total Body Weight (TBW), Body Mass Index (BMI), and gastrointestinal tolerability profiles.

∗**Abbreviations:** RC: Retrospective Cohort; CC: Case-Control; IBD: Inflammatory Bowel Disease; TBW: Total Body Weight; NA: Not Available. *Represents drug-specific ranges (Tirzepatide/Semaglutide).*

The primary focus of these investigations was the impact of GLP-1 receptor agonists—specifically liraglutide, semaglutide, and tirzepatide—on anthropometric outcomes, including total body weight (TBW) and Body Mass Index (BMI). Quantitative data were reported as means or medians, supplemented by standard deviations or ranges where available. Adverse event profiles, including nausea, diarrhea, constipation, and vomiting, were systematically evaluated. The methodological quality assessment for all included studies is detailed in Table S3.

### 3.3 Weight loss effects

The majority of studies reported significant weight loss in patients with obesity and IBD treated with GLP-1 RAs. For example, in the study by Belinchon (2024), patients treated with GLP-1 RAs experienced a median weight loss of −6.2 kg (range: −3.4 to −8.5 kg) after 6 months. Similarly, Clarke (2024) reported a mean BMI reduction of −3.45 kg/m^2^ (SD = 3.47), with a corresponding mean weight loss of 9.27 kg (SD = 8.74) after 12 months of treatment. In Desai (2024), patients with IBD and obesity showed a mean weight loss of −16 lbs (SD = 13) over 18 months, with more pronounced effects observed with tirzepatide (mean = −26 lbs, SD = 20) and semaglutide (mean = −18 lbs, SD = 17). Levine (2024) found a median BMI reduction of −2.4 kg/m^2^, indicating some reduction in body weight in patients with IBD after 12 months, although no significant overall weight loss was reported. Pham (2023) observed a more modest reduction in BMI of −1.8% for liraglutide and −9.8% for semaglutide in patients with obesity and IBD after 12 months, indicating variability in the effectiveness of different GLP-1 RAs. Sehgal (2024) found a mean weight loss of −16 lbs (SD = 7.5) after 3–6 months of GLP-1 RA treatment in adult IBD patients. In contrast, St Pierre (2024) reported a median weight loss of −8.15 kg (range: −15.9 to −2.2 kg) in patients with obesity and IBD over 450 days.

### 3.4 Effect of specific GLP-1 receptor agonists

The effects of specific GLP-1 RAs were explored in several studies. Desai (2024) and Sehgal (2024) observed significant weight loss in patients treated with tirzepatide and semaglutide, with Desai (2024) reporting an average loss of −26 lbs (SD = 20) for tirzepatide and −18 lbs (SD = 17) for semaglutide. On the other hand, Pham (2023) reported a less pronounced reduction in BMI of −1.8% for liraglutide and −9.8% for semaglutide. Levine (2024) found a modest reduction in BMI of −2.4 kg/m^2^, indicating some improvement, but no significant overall weight change. Belinchon (2024), Clarke (2024), and Sehgal (2024) also observed weight loss with varying degrees of effect depending on the specific GLP-1 RA used, with Sehgal (2024) reporting the most moderate weight loss of −16 lbs (SD = 7.5) over a shorter follow-up period.

### 3.5 Follow-up duration and weight loss

Follow-up periods generally demonstrated greater weight loss. For instance, Desai (2024) with an 18-month follow-up observed substantial weight loss, particularly with tirzepatide, compared to studies with shorter follow-up periods such as Belinchon (2024) and Clarke (2024), which reported more moderate weight loss outcomes after 6–12 months. **St** Pierre (2024), with a follow-up of 450 days, also observed substantial weight loss, reinforcing the notion that the effects of GLP-1 RAs on weight loss may increase over time. This suggests that sustained treatment with GLP-1 RAs over longer periods may lead to more pronounced reductions in weight.

### 3.6 Adverse events and weight loss

Common adverse events across the included studies included gastrointestinal symptoms such as nausea, diarrhea, and constipation. For example, Clarke (2024) reported 35 cases of nausea and 10 cases of diarrhea in patients on GLP-1 RAs, while Desai (2024) reported no adverse events in their large cohort. Sehgal (2024) also observed significant gastrointestinal discomfort, with nausea reported in 12 patients. Belinchon (2024) reported mild gastrointestinal symptoms, including nausea and diarrhea in 2 patients each. St Pierre (2024) reported nausea in 11 patients and constipation in 9 patients. These gastrointestinal side effects were commonly reported across studies, highlighting that while GLP-1 RAs promote weight loss, gastrointestinal side effects remain a common concern for IBD patients.

## 4 Discussion

This review article synthesizes the contemporary evidence regarding the therapeutic utility of GLP-1 RAs in addressing obesity within the inflammatory bowel disease (IBD) population. Our findings indicate that GLP-1 RAs represent a promising pharmacological strategy for weight modulation in this clinically distinct cohort, with substantial evidence corroborating their efficacy in achieving significant reductions in total body weight. Nevertheless, the safety profile specifically the incidence and severity of gastrointestinal adverse events necessitates rigorous clinical oversight. These results underscore the urgent requirement for high-quality, prospective research to establish standardized protocols and optimize the integration of incretin-based therapies into the multidisciplinary management of patients with obesity and IBD.

The outcomes from the included studies consistently show that GLP-1 RAs are effective in promoting weight loss in patients with IBD and obesity. For instance, Desai et al. [21] and Sehgal et al. [24] observed significant reductions in body weight among patients treated with tirzepatide and semaglutide, respectively. Sehgal et al. [24] reported a mean weight loss of 16 lbs over a 3–6 month follow-up, while St Pierre et al. [25] documented a median weight loss of 8.15 kg over a longer follow-up period (450 days). These results align with the established role of GLP-1 RAs in promoting satiety and reducing appetite [26], both of which are crucial mechanisms for weight loss in obese patients. The reduction in BMI and TBW observed across studies is significant, highlighting the potential for GLP-1 RAs to help manage obesity in IBD patients, a group who often face challenges with conventional weight loss methods.

Importantly Clarke et al. [20] and Pham et al. [23] also demonstrated similar weight loss outcomes, though to a lesser degree. In these studies, reductions in BMI were observed but with more modest results (mean reductions of 3.45 kg/m^2^ and 1.8% for liraglutide and 9.8% for semaglutide, respectively). These differences may be explained by factors such as baseline obesity severity, medication adherence, and treatment duration. Levine (2024) [22] also found a modest reduction in BMI (mean = −2.4 kg/m^2^), though it did not reach statistical significance. This variability suggests that while GLP-1 RAs are effective for weight loss in the IBD population, the degree of success may vary depending on patient characteristics, type of GLP-1 RA used, and treatment protocols. The variability in weight loss outcomes with GLP-1 RAs in IBD patients can be attributed to factors such as the specific GLP-1 RA used, baseline patient characteristics, disease severity, and treatment duration. Semaglutide and tirzepatide generally lead to more significant weight loss compared to liraglutide, likely due to differences in their mechanisms, with tirzepatide also enhancing glucose-dependent insulinotropic peptide (GIP). Patients with higher BMI or less severe IBD tend to experience greater weight reductions, while active disease flare-ups may reduce treatment efficacy. Longer treatment durations often result in more sustained weight loss, but treatment adherence can vary, with non-adherence due to side effects or cost potentially influencing outcomes.

A primary clinical consideration regarding the administration of GLP-1 RAs in patients with IBD pertains to their gastrointestinal tolerability. The synthesized literature consistently identifies gastrointestinal adverse events as the most prevalent treatment-emergent complications. For example, Clarke et al. [20] reported nausea in 35 patients, while Sehgal et al. [24] found nausea in 12 patients, as well as other adverse effects like diarrhea, constipation, and headache. St Pierre et al. [25] noted nausea in 11 patients and vomiting in 3. These findings highlight the importance of closely monitoring IBD patients for gastrointestinal symptoms when using GLP-1 RAs, as these medications may exacerbate existing GI distress, particularly in individuals with active disease.

While gastrointestinal adverse events were frequently reported, their severity exhibited significant variability across the included studies. Notably, many investigations documented symptoms as clinically manageable, seldom necessitating treatment discontinuation. For example, Desai et al. [21] with a larger cohort, did not report any significant adverse events, suggesting that the safety profile of GLP-1 RAs may depend on factors such as disease activity, comorbidities, and the specific agent used. Importantly, these side effects are well-documented in the broader GLP-1 RA literature, and strategies such as slow dose titration or combination therapies may help mitigate them in IBD patients. Nonetheless, the presence of more serious side effects, such as pancreatitis, in some studies, particularly Sehgal et al. [24] emphasizes the need for caution and patient-specific considerations when prescribing these agents.

Results from this review suggest that GLP-1 RAs hold considerable promise for managing obesity in IBD patients, a group that faces increased risks associated with obesity, such as worse disease outcomes, metabolic syndrome, and reduced quality of life. Given the rising prevalence of obesity in IBD, especially among those with coexisting metabolic disorders, GLP-1 RAs may provide a valuable pharmacological option for weight management in this population. However, the variability in weight loss outcomes and safety concerns, particularly gastrointestinal side effects, indicate the need for personalized treatment approaches. It is essential for clinicians to consider patient-specific factors such as disease severity, comorbidities, and baseline weight when prescribing GLP-1 RAs. Furthermore, additional research is needed to identify the most appropriate GLP-1 RA for individual patients and to assess the long-term safety and efficacy of these agents in IBD populations with varying disease activity.

This study is subject to some limitations that warrant consideration. Notably, the geographical scope of the included literature is heavily skewed, with six of the seven analyzed studies conducted within the United States and only one in Spain. This lack of international diversity significantly limits the global generalizability of our findings, as healthcare infrastructures, dietary habits, and clinical management protocols for obesity and IBD vary substantially across different regions and ethnicities. Furthermore, the reliance on retrospective observational data predominantly from North American clinical settings precludes the establishment of definitive causal relationships and may introduce selection bias. The relatively small number of studies and the inherent heterogeneity in their designs further constrain our ability to perform a quantitative meta-analysis. Future prospective, multi-center trials involving diverse global populations are essential to validate these outcomes and to determine the long-term safety and efficacy of GLP-1 RAs in the broader, international IBD community.

In addition, studies should explore strategies to mitigate gastrointestinal side effects, such as optimizing dosing regimens or investigating the potential benefits of combination therapies. Furthermore, it is essential to assess the impact of GLP-1 RAs on other IBD-related outcomes, including disease remission, symptom control, and quality of life. To enhance the generalizability and robustness of findings, future studies should involve larger, more geographically diverse populations and adhere to standardized methodologies. These efforts will be crucial for advancing our understanding of the long-term clinical benefits and safety profile of GLP-1 RAs in IBD management. Although sex-specific differences in GLP-1 RA response have been suggested in broader metabolic research, the studies included in this review lacked sufficient disaggregated data to perform a sex-based subgroup analysis. Future research should prioritize gender-stratified outcomes to refine personalized treatment protocols.

## 5 Conclusion

Current evidence substantiates the efficacy of GLP-1 Ras most notably semaglutide and tirzepatide in achieving significant weight reduction among patients with IBD. However, the observed variability in outcomes, likely modulated by baseline clinical phenotypes and treatment duration, necessitates further investigation. High-powered, longitudinal studies utilizing standardized methodologies are essential to rigorously validate the safety and efficacy profiles of GLP-1 RAs in this cohort. The inability to evaluate sex-specific effects due to inconsistent reporting in primary studies remains a limitation of the current evidence base.

## Key takeaway clinical messages

• In patients with coexisting obesity and Inflammatory Bowel Disease, clinicians should adopt an integrated management strategy that addresses both metabolic dysfunction and chronic intestinal inflammation.
• GLP-1 receptor agonists are effective for achieving clinically meaningful weight loss in this population, with outcomes influenced by treatment duration and baseline BMI; however, gastrointestinal tolerability requires careful monitoring.
• Weight reduction should be considered a key therapeutic goal in IBD patients with obesity, but robust randomized trials are still needed to confirm long-term safety and clarify their impact on disease activity.

## Ethics approval

Not applicable.

## CRediT author statement

A.J. contributed to conceptualization, methodology, data curation, formal analysis, and writing the original draft. performed the literature search, data curation, validation, and contributed to writing-review and editing. D.D contributed to conceptualization, supervision, project administration, and writing-review and editing.

## Ethical considerations

Not applicable.

## Declaration of artificial intelligence (AI) and AI-assisted technologies

During the preparation of this manuscript, the author(s) used artificial intelligence (AI)–assisted tools exclusively for language refinement and grammar correction to enhance clarity and readability. The authors reviewed and edited the content as needed and take full responsibility for the content of the publication.

## Source of funding

No funding was received for this study.

## Conflicts of interest

None.
